# Age-stratified reference intervals for cerebrospinal fluid total protein in adults undergoing lumbar puncture: A 19-year retrospective study

**DOI:** 10.1097/MD.0000000000051507

**Published:** 2026-11-06

**Authors:** Yuan Tian, Jian Li, Kaijia Chen, Yefeng Hu, Dengke Han, Sha Li, Nan Wang, Yunxiu Huang, Renzhou Li, Jinli Xiao, Jinye Xie, Limin Li

**Affiliations:** a Department of Clinical Biochemistry, Zhongshan City People’s Hospital, Zhongshan, Guangdong, China; b Department of Information Management, Zhongshan City People’s Hospital, Zhongshan, Guangdong, China; c State Key Laboratory of Ophthalmology, Guangdong Provincial Clinical Research Center for Ophthalmic Diseases, Sun Yat-sen University Zhongshan Ophthalmic Center, Guangzhou, Guangdong, China; d Department of General Medicine, The Seventh Medical Center of PLA General Hospital, Beijing, China; e Department of Clinical Laboratory, Zhongshan City People’s Hospital, Zhongshan, Guangdong, China.

**Keywords:** age, cerebrospinal fluid total protein, indirect method, lumbar puncture, reference interval, sex

## Abstract

Using 19 years of retrospective clinical data, we estimated age-stratified reference intervals for cerebrospinal fluid total protein (CSF-TP) in a selected reference-like subset of Chinese adults undergoing diagnostic lumbar puncture. We also examined factors associated with CSF-TP concentrations. A retrospective analysis was conducted using 29,519 CSF-TP test records from patients who underwent lumbar puncture at Zhongshan City People’s Hospital between 2006 and 2025. Of these, 4217 records met the basic age and laboratory criteria; after structured chart review of the clinical history, 253 candidate cases remained. After 2 statistically identified outliers were removed, 251 cases were included. Nonparametric methods were used to estimate CSF-TP reference intervals (P2.5–P97.5) for the overall sample and the 2 age groups, and 90% confidence intervals were estimated using bootstrap resampling. Sex-stratified estimates were considered exploratory. The overall reference interval for CSF-TP in the study population was 18.13 to 73.33 mg/dL. When stratified by age, individuals aged 18 to 48 years had a reference interval of 17.15 to 55.37 mg/dL, while those aged 49 to 85 years had a higher interval of 21.85 to 80.51 mg/dL. A positive correlation was observed between CSF-TP concentration and age (Spearman’s ρ = 0.35, *P* < .001). The median CSF-TP in the 49- to 85-year-old group was 40.9 mg/dL, which was 19.2% higher than the median of 34.3 mg/dL in the 18- to 48-year-old group. In addition, a significant sex difference was found: males had a median CSF-TP of 38.7 mg/dL, which was higher than the median of 34.2 mg/dL observed in females (*P* < .001). Statistically significant associations were also found between CSF-TP and cerebrospinal fluid white blood cell and red blood cell counts, but their explanatory power was limited (white blood cell: Pearson *r* = 0.1684, *r*^2^ = 0.0284; red blood cell: Pearson *r* = 0.1293, *r*^2^ = 0.0167). In this single-center reference-like subset of adults undergoing diagnostic lumbar puncture, CSF-TP increased with age. The age-related reference intervals estimated in this study require direct-method validation and may assist the interpretation of CSF-TP results in similar clinical populations.

## 1. Introduction

Cerebrospinal fluid total protein (CSF-TP) plays a crucial role in the diagnosis, therapeutic monitoring, and prognostic evaluation of neurological disorders.^[1]^ The currently accepted reference interval for CSF-TP – 15 to 45 mg/dL – is widely used worldwide and originates from Merritt and Fremont-Smith’s seminal 1937 monograph *The Cerebrospinal Fluid*.^[2]^ However, recent studies^[3–5]^ have suggested that the upper limit of this reference interval may be too low, particularly for older adults. Relying on a fixed reference interval may lead to the misclassification of age-related variations in CSF-TP as pathological. This may contribute to overinterpretation of albuminocytologic dissociation or barrier dysfunction.^[6,7]^ In addition, the methodologies and population characteristics used to establish the original reference intervals more than 80 years ago differ substantially from those used in modern clinical laboratories and from today’s patient populations. As a result, the applicability of these historical reference intervals has come under increasing scrutiny. Consistent with this, the Clinical and Laboratory Standards Institute EP28-A3c guideline emphasizes that laboratories should establish or verify reference intervals for their intended populations.^[8]^

Two primary approaches exist for defining such intervals: the direct and indirect methods.^[9]^ The direct method is considered the gold standard and requires recruiting and testing a large number of healthy individuals but is costly and operationally complex. Conversely, the indirect method derives reference intervals from existing laboratory data, making it more feasible and cost-effective, particularly for analytes like CSF-TP, where healthy samples are difficult to obtain.^[10,11]^ Although indirect methods may introduce selection bias, they offer a practical alternative for reference interval establishment. The Committee on Reference Intervals and Decision Limits of the International Federation of Clinical Chemistry and Laboratory Medicine recognizes indirect analysis of routine laboratory data as a useful approach for estimating reference intervals.^[11]^ Accordingly, this study applied an indirect method to 19 years of clinical laboratory data to derive hypothesis-generating estimates of age-stratified CSF-TP reference limits in a selected reference-like subset of Chinese adults undergoing diagnostic lumbar puncture.

## 2. Materials and methods

### 2.1. Materials

This retrospective study included data from patients hospitalized at Zhongshan City People’s Hospital who underwent lumbar puncture for CSF-TP testing between January 2006 and April 2025. Raw data were extracted from the hospital’s electronic data platform to construct a research database. The dataset included unique patient identifiers, age, sex, sample number, test items, test results, measurement units, analytical instruments, sample receipt time, and clinical diagnoses. Test items included cerebrospinal fluid (CSF) white blood cell (WBC) count, red blood cell (RBC) count, glucose, and total protein. CSF WBC and RBC counts were matched to CSF glucose and total protein measurements from the same patient. The matched results were required to originate either from the same CSF specimen or from CSF specimens collected within 24 hours of each other.

Data processing was conducted in 2 stages. First, data cleaning involved removing nonnumeric characters from test result fields, validating and correcting original entries, and converting age data to numerical values. Second, records were screened according to age and laboratory criteria: age ≥18 years; complete matched CSF WBC, RBC, glucose, and total protein results; CSF WBC ≤ 5 × 10^6^/L; CSF RBC ≤ 25 × 10^6^/L; and CSF glucose ≥ 2.5 mmol/L. For the reference-interval analysis, if a patient had more than 1 record meeting these basic inclusion criteria, only the chronologically first eligible record was retained; subsequent records were excluded. The retained records then underwent structured chart review. The indication for lumbar puncture and the clinical history were considered to exclude known or suspected conditions that could cause secondary elevations in CSF-TP.

Clinical exclusion criteria covered infectious, inflammatory, and other conditions that could affect CSF-TP. The main categories were central nervous system infections, inflammatory demyelinating diseases, malignant tumors, and relevant cerebrovascular, spinal cord, and peripheral nerve disorders. Representative diagnoses included meningitis, encephalitis, Guillain–Barré syndrome (GBS), chronic inflammatory demyelinating polyradiculoneuropathy (CIDP), multiple sclerosis, subarachnoid hemorrhage, and cervical spondylotic myelopathy. Case selection considered the clinical context and was not based on diagnostic labels alone. Inclusion and exclusion decisions were made case by case through manual review of each patient’s available medical records, including the indication for lumbar puncture, clinical history, admission and discharge diagnoses, neurological findings, available imaging, and laboratory data. Cases were excluded when the overall review identified an active or clinically significant condition that could affect CSF-TP; cases without such evidence were retained. The main steps of case selection are shown in Figure 1; complete diagnostic categories and case counts are provided in Material S1, Supplemental Digital Content 1.

**Figure 1.**
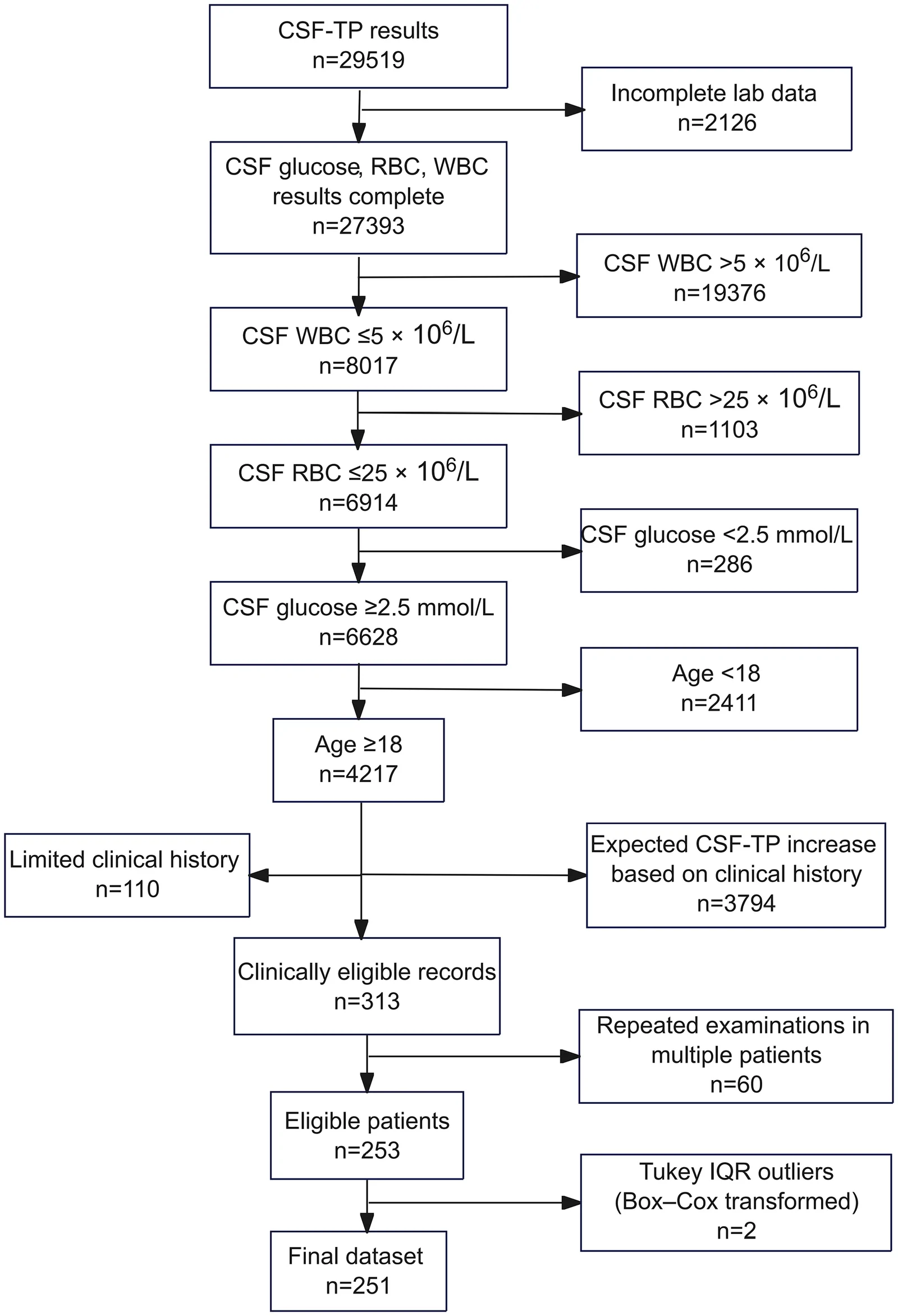
Flowchart of record screening and patient selection for the reference-like subset. CSF = cerebrospinal fluid, CSF-TP = cerebrospinal fluid total protein, IQR = interquartile range, RBC = red blood cell, WBC = white blood cell.

This study was approved by the Ethics Review Committee of Zhongshan City People’s Hospital (approval number: K2025-059). The requirement for informed consent was waived because the study used de-identified, preexisting data.

### 2.2. Instruments and reagents

CSF-TP testing was carried out using the following biochemical analyzer platforms: Siemens ADVIA 2400 (January 2006–April 2025), Roche P800 (November 2011–August 2013), and Roche cobas 8000 (August 2013–April 2025). The Roche systems used their proprietary reagents based on the benzethonium chloride method, while the Siemens system employed the Lidman reagent based on the pyrogallol red method. CSF total protein and glucose levels were measured simultaneously using the biochemical analyzers mentioned above. Because the Roche P800 and Roche cobas 8000 used the same method, their data were combined as the Roche group for the platform comparison.

CSF WBC and RBC counts were performed using the following hematology analyzers: Sysmex XE5000 (January 2006–August 2016), Sysmex XN-10 (August 2016–April 2025), and Mindray BC7500CS (February 2023–April 2025). All specimens were collected, received, and tested according to the laboratory’s standard operating procedures.

### 2.3. Quality control

All testing in this study was conducted in the clinical laboratory of Zhongshan City People’s Hospital, which is accredited under the International Organization for Standardization 15189. Standard operating procedures were strictly followed throughout the testing process. Internal quality control was performed daily, and patient samples were analyzed only after quality control results were verified to be within acceptable limits. The laboratory regularly participated in external quality assessment programs organized by the National Center for Clinical Laboratories under the National Health Commission of China and by the relevant authority in Guangdong Province. Internal comparisons of relevant assays were also performed as required by the laboratory’s quality management system.

### 2.4. Statistical analysis

Statistical analyses and graphical visualizations were performed using R (version 4.3.0; R Foundation for Statistical Computing, Vienna, Austria). The CSF-TP distribution was right-skewed; a Box–Cox transformation was applied before outlier screening. Tukey’s 1.5 × interquartile range rule was then applied on the transformed scale; details of the transformation and outlier identification are provided in Material S2, Supplemental Digital Content 2.

Decision tree analysis was used to assist age stratification, and the main analysis used groups aged 18 to 48 years and 49 to 85 years. The distribution of cutpoints was assessed using 1000 bootstrap resamples, and alternative partitions at 30 and 50 years were compared; details are provided in Material S3, Supplemental Digital Content 3.

For the overall sample and each subgroup, central 95% CSF-TP reference intervals (P2.5–P97.5) were estimated using a nonparametric method. The 90% confidence intervals (CIs) for the lower and upper reference limits were estimated using 1000 bootstrap resamples. Overall and age-stratified reference intervals were the primary results; sex-stratified reference intervals were exploratory estimates. Sensitivity analyses assessed the effects of outlier handling and selected included diagnoses; details are provided in Material S4, Supplemental Digital Content 4.

To compare CSF-TP levels between age and sex groups, the nonparametric Wilcoxon rank-sum test was used. Spearman’s rank correlation was used to assess the association between age and CSF-TP. Separate exploratory record-level Pearson correlation analyses were used to assess the associations of CSF-TP with WBC and RBC counts among records with positive cell counts (n = 25,433 and n = 22,439, respectively). Records with zero or missing cell-count values were excluded, while repeated records from the same patient were retained. CSF-TP distributions between platforms were compared using the Mann–Whitney *U* test. Platform effects were assessed by linear regression after Box–Cox transformation with adjustment for age and sex. All hypothesis tests were two-tailed, with a *P* < .05 considered statistically significant.

## 3. Result

### 3.1. Summary of the study population

Of the 29,519 records, 4217 met the basic age and laboratory criteria. After structured chart review of the clinical history, 253 candidate cases remained. The 3964 excluded records comprised 3794 with relevant conditions, 110 with limited clinical history, and 60 repeat examination records. Two statistically identified outliers were then removed, yielding 251 cases for the main analysis (Fig. 1).

The final cohort consisted of 143 males (mean age: 50.2 ± 18.7 years) and 108 females (mean age: 46.9 ± 18.3 years). There were 124 cases aged 18 to 48 years and 127 aged 49 to 85 years; sample sizes for the age and sex subgroups are shown in Table 1. Included cases were mainly patients with no identified intracranial disease who presented with headache, other pain-related conditions, or vestibular symptoms. They also included patients with motor-neuron-related disorders, mild cognitive impairment, cerebellar ataxia, progressive muscular dystrophy, and selected metabolic or toxic encephalopathies, cerebrovascular conditions, and spine or bone disorders. Diagnostic categories and case counts are provided in Material S1, Supplemental Digital Content 1.

**Table 1:** Cerebrospinal fluid total protein reference intervals by age and sex (mg/dL).

Age (yr)	Overall			Males			Females		
Age, years	P2.5 (90% CI)	P97.5 (90% CI)	n	P2.5 (90% CI)	P97.5 (90% CI)	n	P2.5 (90% CI)	P97.5 (90% CI)	n
18–48	17.15 (16.40–18.81)	55.37 (52.05–56.90)	124	18.86 (17.80–23.63)	55.70 (53.00–57.00)	66	16.57 (16.40–18.50)	51.42 (46.60–56.90)	58
49–85	21.85 (18.20–23.40)	80.51 (72.74–84.40)	127	23.85 (22.10–25.50)	80.44 (72.72–85.40)	77	18.74 (15.20–22.65)	77.94 (61.90–83.70)	50
Overall	18.13 (16.50–19.15)	73.33 (67.55–81.70)	251	20.10 (18.80–23.81)	74.44 (69.87–84.40)	143	16.67 (16.01–18.43)	67.45 (57.93–81.61)	108

P2.5 and P97.5 represent the 2.5th and 97.5th percentiles, respectively. Values in parentheses are 90% CIs for the corresponding reference limits; “n” denotes sample size. Age-stratified reference intervals are the primary results, and both age groups met the CLSI EP28-A3c recommendation of at least 120 individuals per partition for nonparametric estimation.^[8]^ Age-sex subgroups had limited sample sizes; their reference intervals are exploratory estimates and should be interpreted cautiously and confirmed in larger studies.

CI = confidence interval, CLSI = Clinical and Laboratory Standards Institute.

### 3.2. Analysis of factors influencing total CSF protein

CSF-TP concentration demonstrated a modest but statistically significant positive correlation with age (Spearman’s ρ = 0.35, *P* < .001; Fig. 2). Scatter plots revealed an overall increase in CSF-TP levels with advancing age, although a wide dispersion of data points indicated considerable inter-individual variability. Comparisons by sex (Fig. 3A) indicated that males had a higher median CSF-TP level of 38.7 mg/dL, accompanied by a broader distribution, compared to females with a median of 34.2 mg/dL and a more centralized distribution. The difference between sexes was statistically significant (*P* < .001). This age-related trend was further supported by subgroup analysis (Fig. 3B): the median CSF-TP concentration in the 18- to 48-year-old group was 34.3 mg/dL with a symmetrical distribution, whereas the 49- to 85-year-old group showed a higher median of 40.9 mg/dL, with a right-skewed distribution and greater data spread. Pearson correlation analyses showed weak but statistically significant positive correlations between CSF-TP and WBC count (Pearson *r* = 0.1684, *r*^2^ = 0.0284, *P* < .001, n = 25,433; Fig. 4A) and between CSF-TP and RBC count (Pearson *r* = 0.1293, *r*^2^ = 0.0167, *P* < .001, n = 22,439; Fig. 4B). WBC and RBC counts explained only 2.84% and 1.67% of the variation in CSF-TP, respectively. The very small *P* values reflect the large sample size and do not indicate strong clinical explanatory power; these associations alone cannot be used to define new cell-count exclusion thresholds.

**Figure 2.**
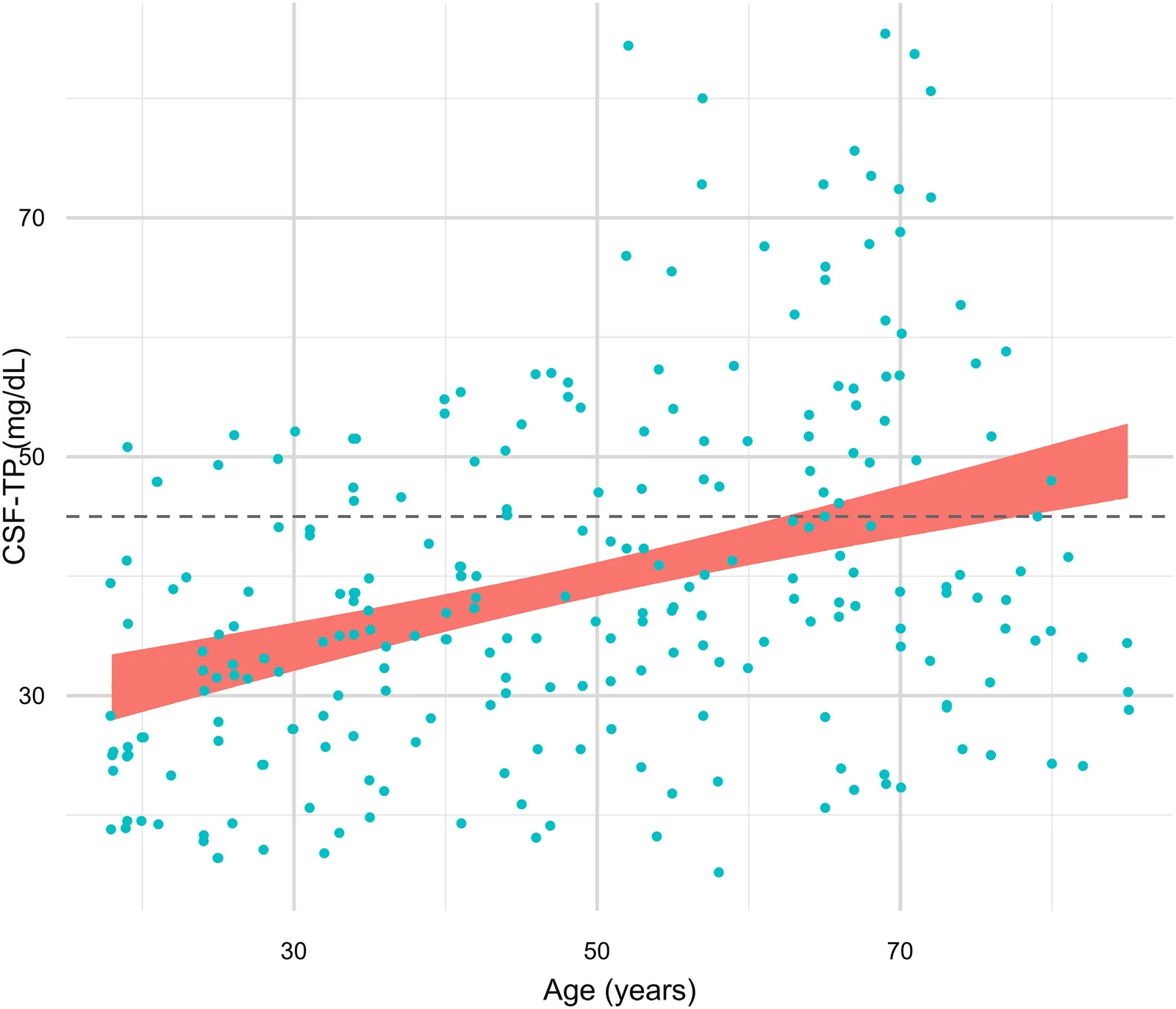
Relationship between age (years) and CSF-TP levels. The trend line represents a linear fit, with the shaded area indicating the 90% CI. The dashed line denotes the conventional upper reference limit of 45 mg/dL. CI = confidence interval, CSF-TP = cerebrospinal fluid total protein.

**Figure 3.**
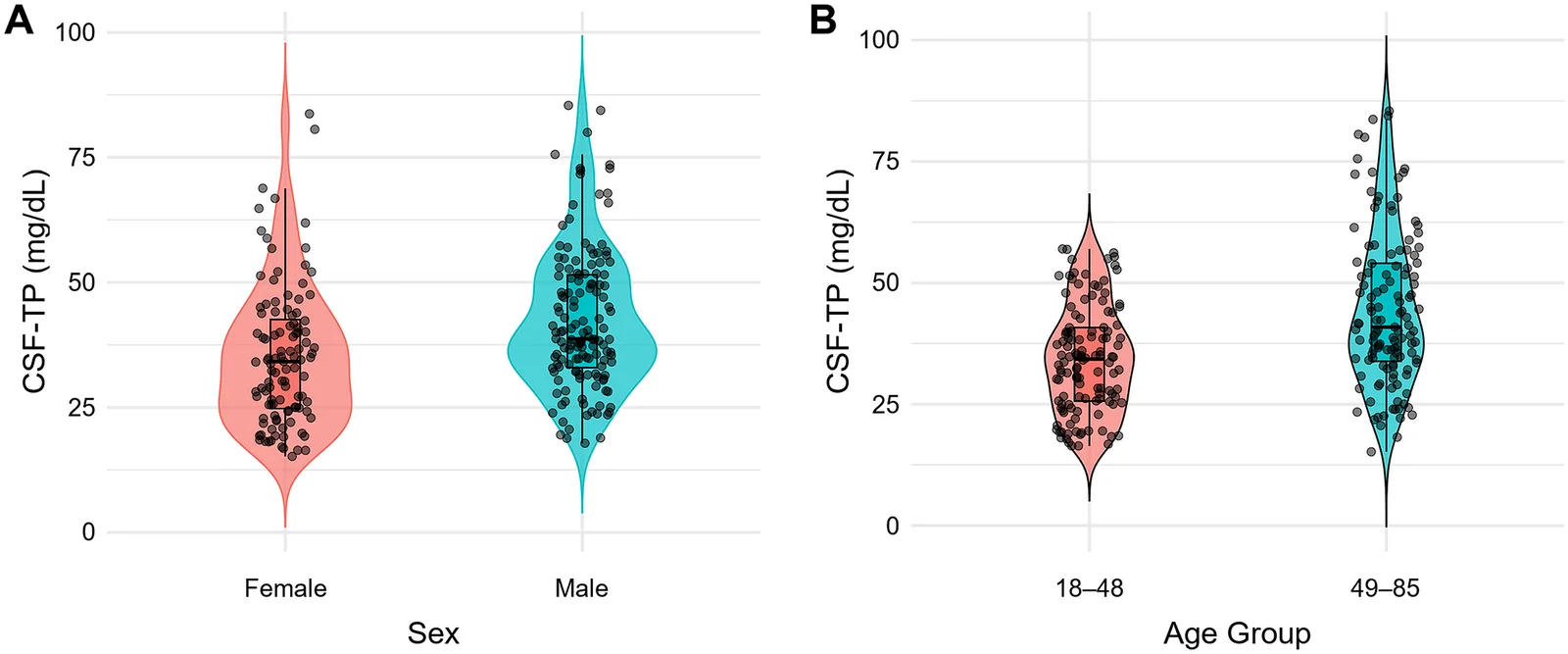
CSF-TP distribution by sex and age group. Distribution stratified by sex (A). Distribution stratified by age group (B). CSF-TP = cerebrospinal fluid total protein.

**Figure 4.**
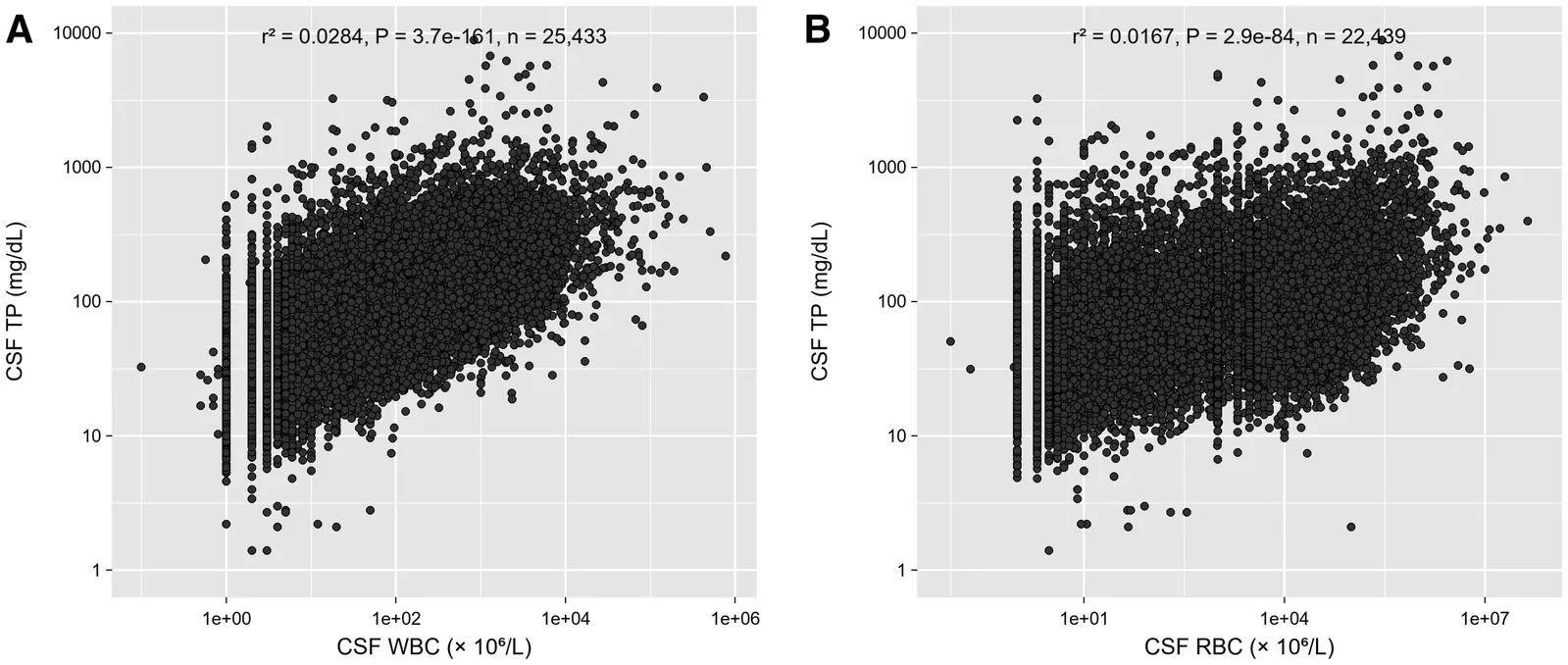
Correlations of CSF-TP with CSF WBC and RBC counts. (A) Correlation between CSF-TP and WBC count (Pearson *r* = 0.1684, *r*^2^ = 0.0284). (B) Correlation between CSF-TP and RBC count (Pearson *r* = 0.1293, *r*^2^ = 0.0167). Both the x- and y-axes in panels “A” and “B” were displayed on logarithmic (log_10_) scales for visualization. CSF = cerebrospinal fluid, CSF-TP = cerebrospinal fluid total protein, RBC = red blood cell, WBC = white blood cell.

There were 208 cases tested on the Siemens ADVIA 2400 and 43 cases on the Roche platforms. Median CSF-TP concentrations were 37.2 and 37.8 mg/dL, respectively. The distributions did not differ significantly (Mann–Whitney *U* test, *P* = .192), and the platform effect was not significant after adjustment for age and sex (*P* = .228).

### 3.3. Establishment of reference intervals

The overall CSF-TP reference interval was 18.13 to 73.33 mg/dL. The reference intervals were 17.15 to 55.37 mg/dL for the 18- to 48-year-old group and 21.85 to 80.51 mg/dL for the 49- to 85-year-old group. Reference limits and their 90% CIs are shown in Table 1. The upper reference limits in both age groups exceeded the conventional fixed upper limit of 45 mg/dL, particularly in the older group.

Reference intervals obtained using alternative age partitions and sensitivity analyses were close to those from the main analysis (Materials S3 and S4, Supplemental Digital Content 3 and 4).

## 4. Discussion

There is currently no widely accepted age-specific reference interval for CSF-TP. In this study, we estimated age-related reference intervals in a selected reference-like subset of Chinese adults undergoing diagnostic lumbar puncture. These intervals require validation by direct methods. Yoshihara et al^[5]^ reported an upper reference limit of 73.2 mg/dL in healthy older Japanese volunteers. This value is numerically close to the overall upper limit of 73.33 mg/dL in the present study. However, the source populations and age distributions differed, and this numerical similarity cannot substitute for direct-method validation.

Previous studies of patients undergoing lumbar puncture for suspected neurological disease have documented a positive correlation between CSF protein levels and age.^[4,12–14]^ The present study also found this association (Spearman’s ρ = 0.35). Dufour-Rainfray et al^[15]^ did not observe a significant age-related increase in CSF-TP among older adults, indicating that findings have not been entirely consistent. The study by Eeg-Olofsson et al,^[16]^ which included participants aged 6 months to 30 years, does not adequately capture age-related physiological changes in older adults. Breiner et al^[17]^ suggested that the widely used upper reference limit of 45 mg/dL may be appropriate only for younger adults, proposing a revised threshold exceeding 60 mg/dL for those over 50 years. Taken together, the present study and other major investigations^[4,5,12,17]^ point to the same conclusion. A fixed upper limit of 45 mg/dL may underestimate normal CSF-TP levels in older adults. This underestimation could increase the false-positive classification of CSF-TP elevation, potentially prompting unnecessary diagnostic evaluation or contributing to misinterpretation in the assessment of neuroinflammatory diseases.^[17–19]^ The physiological basis of age-related increases remains uncertain; reduced CSF turnover and changes in barrier function may contribute.^[20–22]^

In this study, we observed a significant sex difference in CSF-TP concentrations: males had higher values than females. This is consistent with multiple previous studies.^[4,5,23,24]^ McCudden et al^[4]^ suggested endocrine differences and variations in muscle mass as possible explanations. However, the physiological basis of this difference remains uncertain. Parrado-Fernández et al^[25]^ suggested that differences in blood–brain barrier permeability may also be involved. This explanation remains speculative. Based on these observed sex differences, we estimated CSF-TP reference intervals stratified by both age and sex. Because the sample sizes after joint age–sex stratification were small, these estimates should be interpreted as exploratory.

Elevated CSF-TP with a normal or low cell count may provide supportive information in the evaluation of CIDP and GBS, but it must be interpreted together with clinical presentation, disease course, and electrophysiological findings.^[1,6,7,18]^ If a fixed upper limit of 45 mg/dL is used, some mildly increased values may be classified as abnormal. Under the age-stratified limits estimated here, however, such mildly increased values would remain within the corresponding reference interval: 45 to 55.37 mg/dL in patients aged 18 to 48 years and 45 to 80.51 mg/dL in those aged 49 to 85 years. This may change the weight given to CSF-TP elevation as supportive evidence for albuminocytologic dissociation. These intervals do not replace CIDP or GBS diagnostic criteria and should not be used as treatment decision thresholds. CSF-TP may not be elevated early in GBS; a result below the age-related upper limit does not exclude the disease. Because this study did not include a CIDP or GBS diagnostic cohort with appropriate controls, it cannot determine whether these intervals improve diagnostic accuracy or clinical decisions.

We also analyzed correlations between CSF-TP and CSF WBC and RBC counts, which are often used as exclusion criteria due to their potential influence on CSF-TP concentrations. These variables explained little of the variation in CSF-TP, limiting our ability to establish firm exclusion thresholds from the regression results alone. Existing studies vary widely in their exclusion criteria: some exclude all specimens with detectable cells, whereas others allow WBC ≤ 10 × 10^6^/L and RBC ≤ 500 × 10^6^/L.^[3,12]^ McCudden et al^[4]^ used WBC ≤ 5 × 10^6^/L and RBC ≤ 50 × 10^6^/L. Previous studies suggest that the benzethonium chloride and pyrogallol red methods may differ in their response to hemolysis, although experimental conditions and criteria have not been fully consistent.^[4,26]^ In this study, a WBC threshold of ≤5 × 10^6^/L and a more conservative RBC threshold of ≤25 × 10^6^/L were applied to both methods. The WBC criterion was intended to exclude CSF pleocytosis and possible inflammatory conditions. The RBC criterion was intended to reduce the effects of traumatic puncture and blood contamination on CSF-TP. These thresholds were applied as inclusion and exclusion criteria to obtain a relatively homogeneous reference-like subset, rather than as universal cutoffs for analytical interference.

In the available data, CSF-TP distributions did not differ significantly between the 2 assay methods, and the results were similar after adjustment for age and sex. The laboratory maintained internal quality control, participated in external quality assessment, and conducted internal comparisons of relevant assays throughout the study period. However, the platform comparison was based on unpaired clinical samples and did not use parallel measurements of the same specimen. These results, therefore, do not demonstrate complete method equivalence or interchangeability.

A strength of this study is the integration of 19 years of routine clinical laboratory records with structured chart review of 4217 records that met the basic age and laboratory criteria. Selection of the reference-like subset was based not only on laboratory indicators but also on the indication for lumbar puncture and clinical information. The study provides overall and age-stratified reference intervals with 90% CIs for the corresponding reference limits, which may aid age-related interpretation of CSF-TP in similar clinical populations.

This was a single-center retrospective study. All included patients underwent lumbar puncture for clinical indications and were not community-recruited healthy volunteers; selection bias may remain after structured chart review. The reference intervals are most applicable to clinical populations with similar patient sources and testing conditions, and direct-method validation is still needed. Future multicenter prospective studies with predefined inclusion and exclusion criteria could further assess their applicability. Potential ethnic or population effects on CSF-TP remain unproven and require direct comparative studies. The database did not contain complete information on body mass index, vascular risk factors such as diabetes and hypertension, or radiologically confirmed spinal stenosis, so these factors could not be adjusted for. Future prospective studies should collect relevant clinical and imaging data systematically and use multivariable analysis when sample size permits. Paired CSF and serum albumin measurements were also unavailable, preventing calculation of the CSF/serum albumin quotient and further assessment of the relationship between blood–CSF barrier function and CSF-TP. Clinical interpretation should incorporate other CSF indices, neurological signs, neuroimaging, and electrophysiological findings.

## 5. Conclusion

In this selected reference-like subset of adults undergoing diagnostic lumbar puncture, CSF-TP increased with age, and age-stratified upper reference limits exceeded 45 mg/dL, particularly in the older group. These findings support consideration of age when interpreting CSF-TP. The age-related reference intervals estimated here require direct-method validation and local verification against the laboratory method and intended clinical population before clinical use.

## Author contributions

**Conceptualization:** Yuan Tian, Jian Li, Kaijia Chen.

**Funding acquisition:** Yuan Tian, Limin Li.

**Project administration:** Jian Li, Kaijia Chen.

**Data curation:** Yefeng Hu.

**Formal analysis:** Dengke Han.

**Supervision:** Sha Li.

**Investigation:** Nan Wang.

**Validation:** Nan Wang.

**Methodology:** Yunxiu Huang, Renzhou Li.

**Resources:** Jinli Xiao.

**Writing – original draft:** Yuan Tian, Kaijia Chen.

**Writing – review & editing:** Yuan Tian, Jian Li, Jinye Xie, Limin Li.








