# Supplementary material for: Age-stratified reference intervals for cerebrospinal fluid total protein in adults undergoing lumbar puncture: A 19-year retrospective study
Source: Medicine (Baltimore). 2026 Nov 6;105(45):e51507. doi: 10.1097/MD.0000000000051507 (PMC13747588; doi:10.1097/MD.0000000000051507)
Supplement: Supplementary file 1 [file medi-105-e51507-s001.docx]

**Supplementary Material 1. Diagnostic Categories of Cases**

The table below lists the main diagnostic categories, representative diagnoses, and case numbers for the 251 cases finally included after structured chart review. Eligibility was not determined from diagnostic labels alone. For diagnoses with variable clinical severity or disease activity, the inclusion and exclusion criteria were applied case by case through manual review of the available medical records, including the indication for lumbar puncture, clinical history, admission and discharge diagnoses, neurological findings, imaging findings, and laboratory data. Patients with acute or clinically significant cerebrovascular disease, active metabolic or toxic encephalopathy, cervical spondylotic myelopathy, marked spinal canal stenosis, or spinal cord compression were excluded.

| **Handling** | **Diagnostic category and representative diagnoses** | **Cases** |
| --- | --- | --- |
| Excluded | Malignant tumors (e.g., leukemia, lymphoma) | 1,680 |
| Excluded | Acute or clinically significant cerebrovascular diseases (cerebral infarction, intracerebral hemorrhage, stroke, subarachnoid hemorrhage) | 931 |
| Excluded | Hydrocephalus | 251 |
| Excluded | Central nervous system infections (encephalitis, meningitis, brain abscess, neurosyphilis) | 242 |
| Excluded | Metabolic, toxic, and systemic diseases (uremia, shock, sepsis, idiopathic intracranial hypertension) | 174 |
| Excluded | Spinal cord and peripheral nerve diseases (spinal cord injury, myelopathy, cervical spondylotic myelopathy, Guillain–Barré syndrome, chronic inflammatory demyelinating polyradiculoneuropathy, cauda equina syndrome) | 145 |
| Excluded | Epilepsy and functional neurological disorders | 74 |
| Excluded | Demyelinating diseases (multiple sclerosis, neuromyelitis optica spectrum disorder) | 61 |
| Excluded | Central nervous system tumors (meningioma, pituitary tumor) | 60 |
| Excluded | Non-neoplastic intracranial space-occupying lesions | 53 |
| Excluded | Structural abnormalities of the central nervous system | 37 |
| Excluded | Hypoxic–ischemic encephalopathy | 36 |
| Excluded | Neurodegenerative diseases (Alzheimer disease, prion disease, Huntington disease) | 20 |
| Excluded | Cerebrospinal fluid leak | 12 |
| Excluded | Autoimmune and inflammatory diseases (lupus encephalopathy, severe cases of myasthenia gravis) | 7 |
| Excluded | Schizophrenia | 7 |
| Excluded | Previous central nervous system shunt | 4 |
| Included | Headache, pain, and vestibular presentations without identified intracranial disease(headache, dizziness, peripheral vertigo, sciatica, occipital neuralgia, Ménière disease) | 141 |
| Included | Motor neuron disorders without coexisting encephalopathy or other acute neurological disease (motor neuron disease/amyotrophic lateral sclerosis and spinal muscular atrophy) | 13 |
| Included | Other mild or stable neurological conditions (mild cognitive impairment, cerebellar ataxia, hemifacial spasm, and craniofacial dystonia) | 41 |
| Included | Resolved metabolic or toxic encephalopathies without persistent neurological sequelae | 21 |
| Included | Mild spine and bone conditions without myelopathy or spinal cord compression (lumbar degenerative changes, mild cervical degenerative changes) | 14 |
| Included | Selected mild, stable, or resolved cerebrovascular diagnoses (transient ischemic attack, lacunar cerebral infarction, posterior circulation ischemia) | 13 |
| Included | Stable or resolved neuromuscular disorders without CNS involvement (critical illness myopathy, other myopathies, and progressive muscular dystrophy) | 6 |
| Included | Selected ophthalmic conditions (acute angle-closure glaucoma, bilateral optic atrophy) | 2 |
