# Supplementary material for: Age-stratified reference intervals for cerebrospinal fluid total protein in adults undergoing lumbar puncture: A 19-year retrospective study
Source: Medicine (Baltimore). 2026 Nov 6;105(45):e51507. doi: 10.1097/MD.0000000000051507 (PMC13747588; doi:10.1097/MD.0000000000051507)
Supplement: Supplementary file 2 [file medi-105-e51507-s002.docx]

**Supplementary Material 2. Supplementary Methods**

**Box–Cox Transformation and Outlier Identification**

The 253 candidate cases had a right-skewed distribution of cerebrospinal fluid total protein (CSF-TP). A Box–Cox transformation was therefore applied before outlier screening. The transformation was as follows:

*y(λ) = (y^λ^ − 1) / λ, λ ≠ 0*

*y(λ) = ln(y), λ = 0*

In this formula, y is the original CSF-TP concentration. The maximum likelihood estimate of λ was −0.0154. This value is close to 0, so the transformation is close to a logarithmic transformation.

Tukey’s rule was applied once on the transformed scale. Values below Q1 − 1.5 × IQR or above Q3 + 1.5 × IQR were identified as statistical outliers. Q1 and Q3 are the first and third quartiles. The interquartile range (IQR) is the difference between them.

Two cases were identified. Their original concentrations were 9.8 and 181.7 mg/dL, accounting for 0.79% of the candidate cases. After removing them, 251 cases remained.
