# Supplementary material for: Age-stratified reference intervals for cerebrospinal fluid total protein in adults undergoing lumbar puncture: A 19-year retrospective study
Source: Medicine (Baltimore). 2026 Nov 6;105(45):e51507. doi: 10.1097/MD.0000000000051507 (PMC13747588; doi:10.1097/MD.0000000000051507)
Supplement: Supplementary file 3 [file medi-105-e51507-s003.docx]

**Supplementary Material 3. Age Cutpoints and Alternative Age Partitions**

The single-split decision tree analysis identified a split at 47.5 years. Across 1,000 bootstrap resamples, the median cutpoint was 46.5 years (IQR, 39.0–51.5 years), indicating uncertainty in the exact cutpoint. Only 3 cases were aged 48 years, and assigning these cases to the younger age group produced reference limits that were nearly identical to those obtained using the tree-defined split. For practical clinical grouping, we therefore used the boundary between 48 and 49 years, resulting in groups aged 18–48 years and 49–85 years.

We also compared alternative partitions at 30 and 50 years. The results are shown in the table below.

| **Age partition** | **Age range (years)** | **n** | **Reference interval (mg/dL)** |
| --- | --- | --- | --- |
| Main analysis | 18–48 49–85 | 124 127 | 17.15–55.37 21.85–80.51 |
| Alternative partition, 50 years | 18–49 50–85 | 128 123 | 17.22–55.33 21.82–80.57 |
| Alternative partition, 30 years | 18–29 30–85 | 52 199 | Reference intervals were not estimated for both partitions |

Note: The reference intervals from the 50-year alternative partition were close to those from the main analysis. In the 30-year partition, the 18–29-year group had only 52 cases. This number is below the sample size recommended by the Clinical and Laboratory Standards Institute (CLSI) EP28-A3c guideline, that is, at least 120 individuals per partition. We therefore did not report stratified reference intervals for this partition.
