# Supplementary material for: Age-stratified reference intervals for cerebrospinal fluid total protein in adults undergoing lumbar puncture: A 19-year retrospective study
Source: Medicine (Baltimore). 2026 Nov 6;105(45):e51507. doi: 10.1097/MD.0000000000051507 (PMC13747588; doi:10.1097/MD.0000000000051507)
Supplement: Supplementary file 4 [file medi-105-e51507-s004.docx]

**Supplementary Material 4. Additional Sensitivity Analyses**

Two sensitivity analyses were performed. The first kept the two statistical outliers. The second excluded 13 patients with motor-neuron-related diagnoses. The second analysis was performed because previous indirect-method studies of CSF-TP reference intervals did not handle these diagnoses in exactly the same way. Both analyses used the same age groups and the same non-parametric method as the main analysis. The results are shown in the table below.

| **Analysis** | **n** | **Overall** | **18–48 years** | **49–85 years** |
| --- | --- | --- | --- | --- |
| Main analysis | 251 | 18.13–73.33 | 17.15–55.37 | 21.85–80.51 |
| Keeping the two statistical outliers | 253 | 17.89–74.97 | 16.83–55.36 | 21.85–83.16 |
| Excluding 13 patients with motor-neuron-related diagnoses | 238 | 18.08–72.80 | 17.10–55.00 | 21.68–80.06 |

Note: Values are the 2.5th to 97.5th percentiles, in mg/dL. After excluding the 13 cases, the 18–48-year and 49–85-year groups had 121 and 117 cases, respectively.
